# Community Health Nursing in Iran: A Review of Challenges and Solutions (An Integrative Review)

**DOI:** 10.3389/fpubh.2022.899211

**Published:** 2022-06-27

**Authors:** Aazam Hosseinnejad, Maryam Rassouli, Simin Jahani, Nasrin Elahi, Shahram Molavynejad

**Affiliations:** ^1^Student Research Committee, Nursing and Midwifery School, Ahvaz Jundishapur University of Medical Sciences, Ahvaz, Iran; ^2^Cancer Research Center, Shahid Beheshti University of Medical Sciences, Tehran, Iran; ^3^Nursing Care Research Center in Chronic Diseases, Ahvaz Jundishapur University of Medical Sciences, Ahvaz, Iran

**Keywords:** community health nursing, nursing problems, challenges, solutions, Iran

## Abstract

**Background and Objective:**

In recent decades, nursing has witnessed many changes in Iran. Despite the numerous advances in nursing, the health system faces many challenges in community health nursing. This study aims to review the challenges in community health nursing in Iran and provide an evidence-based solution as well.

**Materials and Methods:**

This article is an integrated review of the literature regarding the challenges in community health nursing published between 2000 and 2021 in the databases Scopus, Medline, Cochrane Database of Systematic Reviews, Science Direct, Google Scholar, Scientific Information Database (SID). After performing searches, 20 articles were selected and studied. Data analysis was done using Russell approach (2005).

**Findings:**

The results of this study were summarized in 6 themes consisting of challenges in community health nursing education, practical challenges in community health nursing, policy-making challenges in community health nursing, management challenges in community health nursing, and infrastructural and cultural challenges. Solutions were also proposed to address each of the above issue.

**Conclusions:**

The results of the study showed that diverse challenges exist in community health nursing in Iran, considering that community health nurses play an important role in providing primary health care and community-based care. In order to solve these challenges, the authors have some recommendations: modifying the structure of the health system with the aim of moving toward a community-oriented approach from a treatment-oriented one, developing laws to support community health nurses, creating an organizational chart for nurses at the community level, modifying nursing students' training through a community-based approach, and covering community-based services and care under insurance.

## Introduction

An examination of nurses' status and position in the service provision system around the world shows that nurses constitute the largest group of health care workers (1, 2). Community health nurses are a major link between the community and health institutions. They are able to understand and interpret the needs of the society and the objectives of health policymakers (3, 4). In addition, community health nurses have an excellent position and status for addressing many challenges in the health system including immigration, bioterrorism, homelessness, unemployment, violence, obesity epidemic, etc. (1, 2, 5). From the perspective of the World Health Organization (WHO) and the American Nursing Association, community health nursing is a special area of nursing that combines nursing skills, public health, and a part of social activities with the aim of promoting health, improving physical and social condition, and rehabilitation and recovery from diseases and disabilities (6, 7). Considering the importance of nursing services in the health service provision system and their role in universal health coverage, in the 66th Session of the health ministers of WHO Regional Office for the Eastern Mediterranean, much attention was devoted to developing plans for improving and strengthening community health nursing (8).

In all developed countries, community health nursing has had a significant growth in the health care system (9). In Canada, the community-based community health nursing has been established in 1978, aiming to maintain and promote the health of individuals, families, and communities. It also participates in the family physician and primary health care delivery (10). In some European countries, including Norway, Finland, the United Kingdom, Ireland, Sweden, and France, community health nurses have replaced physician-centered and hospital-centered approaches, providing health services for the members of the community (4, 9, 11–13). A study conducted by WHO on community health nursing's status in some less developed and developing countries (Bangladesh, Indonesia, Nepal, Cameroon, Senegal, Uganda, Guyana, Trinidad and Tobago) shows a lack of commitment and the low capacity of the policymakers to implement global and regional political tools regarding community health nursing, although most of the countries under study had a basic and operational framework for the optimal activity of community health nurses. On the other hand, only 6% of the community health nurses in these countries worked in the field of health promotion, disease prevention, and rehabilitation care, while this sector is supposed to be their main field of activity. The existing barriers preventing community health nurses from playing their role in developing countries include the lack of consensus in the realm of community health nursing practice, the lack of necessary coordination for inter-professional activities, few job opportunities for community health nurses, insufficient recognition of community health nursing, and great emphasis on clinical care in health centers (6). In Asian countries, including Japan, China, and Malaysia, community health nurses play a key role, too, focusing on the assessment of community health needs, health care delivery, and health promotion (9, 14, 15).

Iran is a populated country in the Eastern Mediterranean region, where health services are provided at public, private, and charity sectors (16, 17). In Iran, since 1958, behavioral and social sciences have been included in the nursing program as a major part of its curriculum. Then, in 1986, the disciplines of community-based and community-oriented nursing were considered by educational policymakers, followed by the inclusion of community health nursing and epidemiology courses in the undergraduate curriculum. Community health nursing program is developed in line with health-oriented policies and focuses on community health. Graduates of this field work in different settings of community by combining the nursing science with other health-related sciences and evidence-based practice (18, 19). Due to its focus on health promotion, the position of this discipline in the country's health system is very crucial, and is a major contributor to directing the community toward the 20-Year National Vision and in an ideal position to address the countless challenges against the health system (2). However, the role of nurses in Iran has not made significant progress and is limited to providing services in medical centers (20), because the viewpoint and the attitude of most Iranian health authorities is based on the employment of nurses in the secondary level of prevention, i.e., clinical care in hospitals (2). Therefore, hospitals are the most common setting for community health nurses' activities (21). Comprehensive health centers are also managed to provide health services by the workers with bachelor's and associate's degrees in family health, environmental health, occupational health, and disease control, as well as midwives. These services are provided sporadically in health service centers (18) and no effective strategies tailored to the needs of the community are adopted in order to provide care (22).

It is noteworthy and interesting that also in the family physician team, no position has been defined for community health nurses and most of the Iranian health authorities believe that nurses cannot provide significant health services (18). However, the community health nurse can be a complementary project in the family physician program and even make up for its shortcomings. This can help the government understand the *health for all* as a goal, the proof of which is the presence of nurses in blood pressure screening program in 2012 (23). On the other hand, numerous studies indicate community health nurses' abilities and their key role in identifying health needs and promoting community health (24–28). Although the education and training of community health nurses is costly for the government, their expertise is not utilized. At present, the services of community health nurses in Iran are mainly provided at the third level and at hospitals, because no position is defined for them in comprehensive health centers (18, 29). In other words, they have no defined job position to work in this field, although in the curriculums, the future job status of this discipline is designed (30). Therefore, one of the most important infrastructural issues is to create a position and a job description in the organizational chart for community health nurses in comprehensive health centers (31).

A brief review shows that studies in Iran have mostly focused on the challenges of community health nursing education and barriers against home care (1, 16, 18, 19, 32–34) and other aspects of community health nursing have rarely been studied. In addition, no study has been conducted to offer solutions for addressing the challenges of community health nursing. Although other studies have been conducted in different cultures and contexts, an integrated review of them can help identify and eliminate present barriers with the aim of facilitating future planning and policymaking to enhance the status of community health nursing. Therefore, by conducting an integrated review, the present study aimed to identify the challenges of and barriers against community health nursing and the strategies to address them.

## Methodology

This is an integrative review study on the challenges of community health nursing and the related solutions. The integrated review of literature is the summarization of previous studies by extracting the study results. This method is used to evaluate the strength of scientific evidence, identify gaps in current research, detect the needs for future research, create a research question, identify a theoretical or conceptual framework, and explore the research methods that have been successfully used. The integrative review study is based on Russell model which consists of 5 steps as follows: (1) formulating the research problem, objective, and question, (2) collecting data or searching through articles, (3) evaluating data, (4) data analysis, (5) interpreting and presenting the results (35).

### Formulating the Research Problem, Objective, and Question

Considering the items discussed in the introduction, this study is conducted to determine the challenges of community health nursing in Iran and the related solutions. Two key questions guiding the review process include “What are the challenges of the community health nursing discipline in Iran?” and “What are the solutions to address these challenges?” Answering these two key questions will help detect the challenges of community health nursing, propose solutions to address them, and promote the community health nursing discipline.

### Collecting Data or Searching Through Texts

In this study, the target population consisted of all the studies (articles and dissertations) that had been conducted in the field of community health nursing regarding its challenges, barriers, and solutions, the full texts of which were accessible. Available resources, including all the studies on the challenges of community health nursing, were reviewed in this study. A comprehensive search was done through the databases Medline, Scopus, Cochrane Database of Systematic Reviews, Science Direct, Google Scholar, and Scientific Information Database (SID) for the papers published between 2000 and 2021 in eligible English or Farsi journals.

The keywords that were searched consisted of community health nurse, community-based nursing, public health nurse, nursing challenges, nursing position, and primary health care. The keywords were investigated both separately and in combination with each other (Table 1). Finally, after preforming the search, 142 published articles were identified.

**Table 1:** Search strategy.

**Databases**	**Searched outcomes**	**Inclusion criteria**
•MEDLINE • Science Direct • Scopus • Google Scholar • Cochrane Library • CINAHL • Scientific Information Database	•Community Health Nurse • Community Based Nursing • Public Health Nurse • Nursing Challenges • Nursing position • Primary health care • Universal Health Coverage • Search terms were specified with the Boolean expression *AND(work OR job)*	•Peer-reviewed publications • January 2000- December 2021 • Being written in either English or Persian • Containing the keywords or their equivalent in the title or abstract of the article
Results database search after exclusion of duplicates	77	**Exclusion criteria** • Not accessing the original paper and the information on its methodology • Being written in other languages
Results manual search	2	
Total identified studies	79	
Removed based on title or abstract	24	
Removed after in-depth reading	35	
Final selection of included articles	20	

### Data Evaluation

The relevant articles were evaluated based on the title, abstract, text, as well as the inclusion and the exclusion criteria. The inclusion criteria for the studies consisted of the following: (1) examining the challenges of and barriers against community health nurses and its position, (2) containing the keywords or their equivalent in the title or abstract of the article (3) Being written in either Farsi or English. The exclusion criteria included the following: (1) not accessing the original paper and the information on its methodology, (2) being written in other languages, (3) being irrelevant to the research question. It is noteworthy that in this study, there were no limitations in terms of research method, so that the results of various studies could be used.

#### Selecting Studies

After doing the systematic search, the studies related to the search keywords were found. After removing the duplicate titles (79 articles), the title, the abstract, and the full text of the studies were reviewed by the research team, and the inclusion and the exclusion criteria were applied. Twenty-four articles were excluded due to being irrelevant and 55 articles entered the screening stage, 20 of which were excluded. Then 35 articles were examined regarding eligibility and, finally, 20 articles were included in the study. The studies were selected by a research team consisting of two nursing professors (faculty members of Ahvaz Jundishapur University of Medical Sciences and Tehran's Shahid Beheshti University of Medical Sciences) and one nursing PhD student (Ahvaz Jundishapur University of Medical Sciences). Furthermore, the research team came to a consensus through more discussion regarding the points of disagreement.

### Data Analysis

At this stage, the articles were reviewed separately. Finally, 20 articles related to the purpose of the study were reviewed and analyzed. Each article was read completely and the results of the studies were extracted from them. After extracting the results of the articles, their results and statistical analysis were compared. The results with the highest frequency in these articles were further interpreted in the next phase.

### Interpreting the Data and Publishing Information

At this stage, according to the analysis of the related studies, their comparison, and the data frequency, the following items were extracted.

## Results

### Search Results

**Figure 1:**
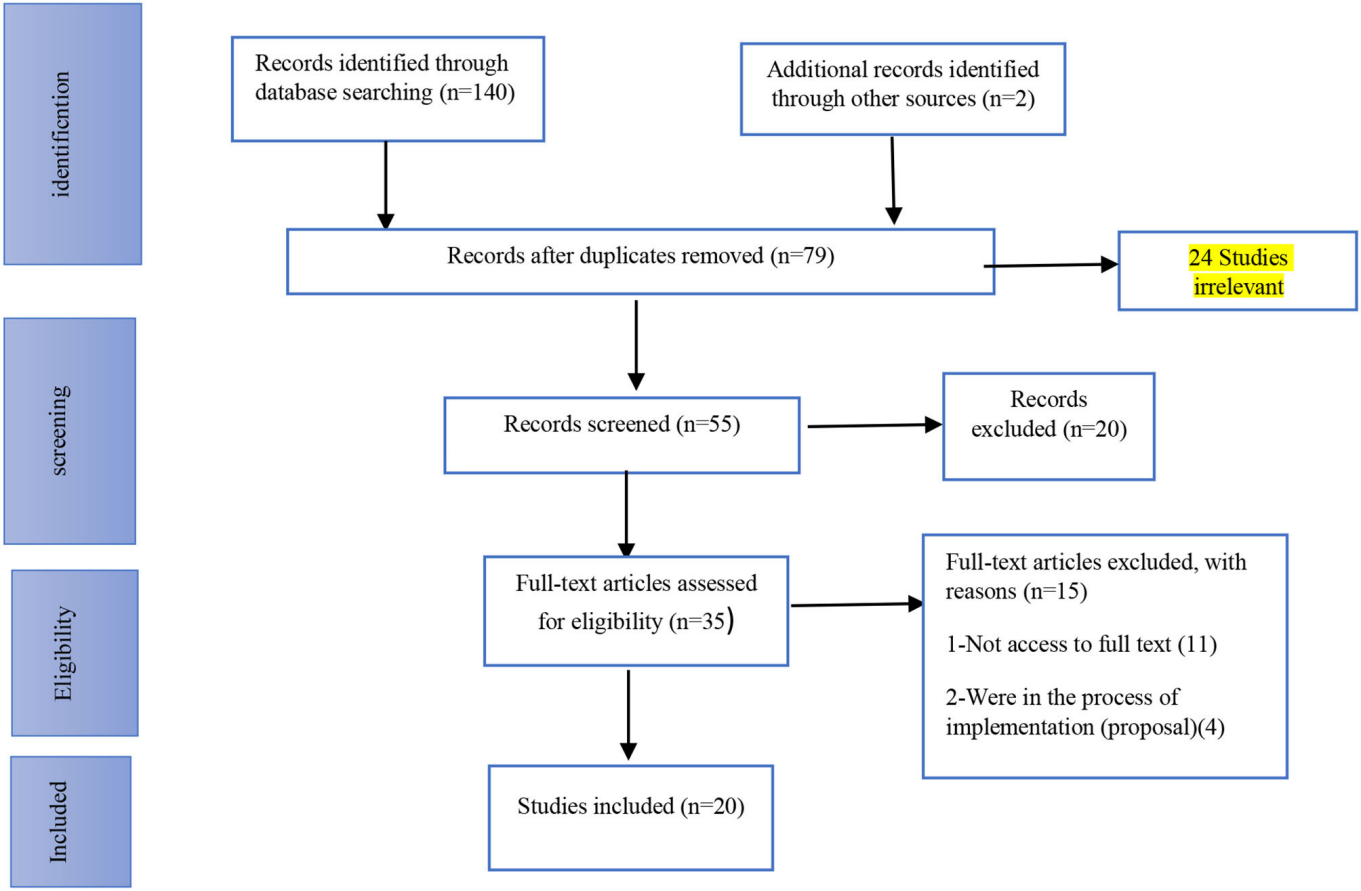
PRISMA flowchart for search strategy and results.

After eliminating the duplicate articles (*n* = 79), 55 studies entered the screening phase and their titles and abstracts were evaluated. In total, 35 studies were included in the selection phase, and 20 remained in the study (Figure 1). Twenty articles met the inclusion criteria and were included in the final analysis. The details are displayed in Table 2.

**Table 2:** A summary of the critical review of previous Iranian and foreign studies.

**No**	**Authors (Year)**	**Title**	**Target group, sample size**	**Important results and interpretations**
1	Laurant et al. (36)	Replacing physicians with nurses in PHC	Systematic review *N* = 18	The care provided by nurses has similar or better health outcomes than the care provided by physicians. Findings showed that primary health care (PHC) directed by nurses reduced mortality in certain groups of patients. There was an increase in patients' satisfaction and quality of life compared to when care was provided by physicians. Nursing consultations were longer and the number of patient's follow-ups was higher with nurses than with physicians. Therefore, since the cost of hiring a nurse is lower than that of a physician, health nurses can be employed instead of physicians.
2	WHO (6)	Study on the status of community health nursing in 22 countries	Cross-sectional study *N* = 432	In African and Southeast Asian countries working condition for community health nurses was poor and unsafe, and lacked resources and the opportunities for the development and the enhancement of the profession. However, in the United States, health care institutes were well-equipped for community health nurses. The findings of the study showed that less than 10% of community health nurses fulfilled the roles regarding health promotion, disease prevention, and client consultation. Therefore, according to this fact, community health nurses less frequently participate in community-based activities
3	Kemppainen et al. (13)	Nurses' role in health promotion practice	Integrated review study *N* = 40	The vague and unclear concept of *health promotion* for nurses, their lack of skills and knowledge of performing health promotion activities, the lack of resources, and insufficient time are important challenges in the field of health promotion. Organizational culture is a key factor in nurses' activities in the field of health promotion. Managers in the organization should value health promotion activities, play a supportive role for nurses in this regard, provide sufficient resources and facilities to nurses for their practice, and continuously train nurses according to the health needs of the community
4	Heydari et al. (18)	The position of community-based nursing in Iran	Qualitative content analysis study *N* = 20	Important challenges in providing community-based nursing include not defining clear job descriptions for community health nurses, the lack of an effective management system to plan the use of community nursing services, the lack of a position for community health nurses in the family physician team, and people's distrust of nurses' capability to provide prevention services. One solution to overcome the challenges is creating an appropriate infrastructure (creating job opportunities, changing policymakers' attitude toward community-based nursing, and preparing the community to accept community-based nursing services)
5	Heidary et al. (2)	The position of nursing in the health service delivery system in Iran	Traditional review study	The main challenge is not being aware of nurses' capabilities in the field of health promotion and prevention, resulting in the very low activity of nurses in health care centers, which is why most nurses work in hospitals (the second level of prevention). Services provision settings for nurses are very limited in the community. Therefore, making reforms in the Iranian health system is necessary. Nursing officials should also work to promote and explain the position of nurses in the health system and prepare community health nursing students for the provision of services at the community level
6	Valaitis et al. (37)	The educational needs of community health nurses regarding the standards of community health nursing practice	Descriptive study (*N* = 1344)	Due to the increased cost of hospital-based care and the important role of community health nurses in health promotion and disease prevention, health system policymakers and decision makers should support this group by allocating sufficient resources to them in order to address their educational needs. Community health nursing instructors and teachers should also adjust their educational content to the needs of students and develop a continuing improvement educational program for community health nursing graduates
7	Philibin et al. (12)	Public health nurse's role in changing the community	Qualitative content analysis (*N* = 25)	The scope of public health nurses' practice is so wide that it includes comprehensive clinical care. This variety of responsibilities often leads to an increased workload and will hinder their practice in the field of health promotion. Therefore, it is recommended to redefine the roles of public health nurses with emphasis on its specialization, because public health nurses play a valuable role in providing community health services
8	Yuan et al. (9)	Community health nursing in China: status, challenges, and the strategies for its development	Descriptive study	The management of comprehensive health centers by physicians instead of community health nurses and the absence of community health nurses at high levels of policymaking hinders their effective practice. Low salary paid by community health centers is another challenge that has led to fewer nurses working in these centers than in hospitals. Therefore, it is necessary to acquaint health policymakers with the importance of community health nursing, to develop laws in support of these nurses, and to allocate sufficient funds to provide community health nursing services
9	Ildarabadi et al. (32)	Nursing students' perception of community health nursing education	Grand theory study (*N* = 14)	Nursing students' poor perception of community health nursing apprenticeship (a chance to rest and have fun, easy and simple content) and their treatment-oriented perspective lead to reduced productivity of community health nursing courses, and as a result, they don't have the required competency for practice at the community level after graduation. Therefore, it seems necessary that instructors correct the approach to community health nursing education
11	Schveitzer et al. (24)	Challenges of nursing in universal health coverage	Systematic review (*N* = 30: 29 qualitative studies, one quantitative study)	Nursing challenges for universal health coverage are related to education and preparation. Therefore, educating nurses is important for their effective performance in providing health services. The clear definition of nurses' roles in primary health care, interdisciplinary and multidisciplinary collaboration, community empowerment, technological advancements, professional communication with care seekers, and providing comprehensive and holistic care are needed to overcome the challenges
12	Mofrad et al. (38)	A comparative study of nursing role and position in the health system of Iran and the selected countries	Comparative study (*N* = 18 countries)	The role and the position of nurses in the Iranian health system have a much lower status compared to the selected countries, and despite the huge funds spent on nursing education at specialized levels, no position is defined for them in the health system promotion and most of them are recruited in hospitals. The preventive role of nurses in Iran is so insignificant that it can be said that such a role does not exist. Therefore, it is necessary for the policymakers to define the role and the position of nurses in the primary health care system
13	Kabayama et al. (14)	The role of community health nurses in long-term projects of preventive healthcare in Japan	Descriptive study	Community health nurses have the necessary knowledge and skills to work at the three levels of prevention. Community health nurses play a key role in providing care for communities with older population such as Japan In Japan, the demand for community health nursing services has increased due to its cost-effectiveness
14	Heravi et al. (19)	Nursing students' perception of apprenticeship in the community health nursing setting	Qualitative content analysis (*N* = 19)	The inadequacy of nursing instructors, the inefficiency of the executive management of apprenticeship, and the theory-practice gap are the challenges of community health nursing education. Therefore, the students' clinical education should be tailored to the needs of the community. These challenges should be considered to improve the quality of apprenticeship programs while planning them
15	Yazdani et al. (21)	An analysis of the healthcare system policymaking in Iran regarding professional nursing	Qualitative content analysis (*N* = 6)	Policymaking in Iranian health system is inappropriate in the field of nursing profession and factors such as the lack of mutual interaction among nursing sectors or other institutions of health care system, the unwillingness of these institutions to explore nursing, and nurses' lack of involvement in the policymaking in this area due to conflicts of interest. The development of disciplines parallel to nursing and the unclear scope of nursing practice in the field of health care services are among the other important challenges
16	Nkowane et al. (39)	Promoting the role of community health nurses for universal health coverage: a performance measurement of community health nursing in 13 countries	Cross-sectional study (*N* = 13 countries)	Community health nurses play an important role in universal health coverage. The performance of community health nurses requires the commitment of policymakers and managers to invest in the development of this profession. These countries had functional frameworks for the education, management, and provision of their services. These countries had appropriate supportive and motivational packages to retain community health nurses in health centers. Few community health nurses (15%) had received interdisciplinary training. Most community health nurses offered a variety of roles in health centers, for about half of which they had not received appropriate training
17	Shahshahani et al. (40)	A study on the status of health care delivery system in Iran	Triangulation study *N* = 15 stage 2 *N* = 64 stage 3	Considering the role of nursing in community health, it is recommended that appropriate planning be done by the health system policy makers in order to create a position in the health sector. The findings of this study showed that the biggest change in Iranian nursing is the provision of services and community-based activities
18	Gultekin (41)	The status quo of public health nursing in Turkey, the related issues and solutions	Descriptive study	Despite the fact that there are PhD courses in public health nursing in Turkey, the community health centers are run by midwives and few public health nurses work in these centers. Therefore, it is necessary to improve the personal rights of public health nurses, restore their occupational identity, and improve the conditions for providing comprehensive health care. The concept of community nursing and home care needs the support of policymakers, and the formulation and reform of laws
19	Heydari et al. (16)	The barriers against home care services in Iran	Qualitative content analysis (*N* = 17)	The cultural aspects of the Iranian community should be considered for developing a position for home care and the integration of nursing into the health system. Infrastructural problems such as the lack of executive protocols, the lack of insurance coverage and interdisciplinary issues should be solved. Treatment-based approaches, the prioritization of hospital care, and the single-dimensional management of the health system are among the other challenges in community-based nursing
20	Hossein Nejad et al. (31)	The requirements for developing community health nursing position in the PHC system in Iran	Qualitative content analysis (*N* = 24)	One of the requirements for creating a position for community health nursing in the primary health care system of Iran is focusing on the dimensions *creating a transparent framework for community health nursing practice, promoting community health nursing education and training for practice in primary health care system and community settings, seeking support, strengthening cooperation and engagement among the key stakeholders of the primary health care system, changing policies and the structure of the health system*, and *focusing on the deficiencies of the health system*. These policies, if properly and correctly implemented in the socio-cultural context and background of the community based on the primary health care system and in line with the efforts and policies of the health system, can help policymakers pay more attention to the challenges that hinder community health nursing practice in the health system

The 20 remaining studies were published between 2010 and 2021. Five of them were review studies (systematic, meta-synthesis and integrated reviews). Seven reviews were of qualitative type (grounded and content analysis), seven reviews were cross-sectional descriptive, and one was a comparative study. Eighteen reviews were published in English and two were published in Farsi. The majority of the reviews investigated the position and the role of community health nursing in the health service delivery system and its challenges. About 40% of the reviews are related to the studies on the situation of community health nursing and the barriers against its provision in Iran. Two reviews have studied community health nursing education in Iran, and seven reviews proposed strategies to solve the challenges of community health nursing.

### Findings

By analyzing the reviews, six themes emerged in the field of community health nursing challenges, and six other themes concerned the strategies to overcome the challenges. The details are displayed in Table 3.

**Table 3:** The challenges of community health nursing and the identified solutions.

**Authors**	**Challenges**
	**Community health nursing training**
Karimi et al. (1), Ildarabadi et al. (32), Heravi et al. (19), Heydari et al. (16), Shahshahani et al. (40), Hosseinnejad et al. (31), Cheraghi et al. (42)	1. Input 2. Process 3. Output
	**Community health nursing practice**
Yazdani et al. (21), Hosseinnejad et al. (31), Heydari et al. (16), Heydari et al. (18)	Not defining a position for community health nursing service delivery Recruiting community health nurses only at hospitals and treatment settings No clear job descriptions for community health nurses
	**Policymaking in the field of community health nursing**
Hosseinnejad et al. (31), Yazdani et al. (21)	Lack of interaction between nursing managers and the policymakers of the Ministry of Health Not involving nurses in policymaking regarding their discipline
	Policymaking in community health nursing
Heidary et al. (2), Heydari et al. (18), Shahshahani et al. (40)	Managerial measures Not being acquainted with community health nursing profession
	**Infrastructure**
Hosseinnejad et al. (31), Heydari et al. (16), Barasteh et al. (29), Heydari et al. (16)	Insurance coverage and funding Executive protocols Interdisciplinary cooperation
	**Culture**
Heydari et al. (16), Heydari et al. (18)	Public distrust of and negative attitudes toward the capabilities of non-physician experts
	**Solutions**
	**Community health nursing training**
Heravi et al. (19), Alizadeh et al. (43), Gimenes et al. (44), Karimi et al. (1), WHO (6), Barasteh et al. (29), Cheraghi et al. (42), Ildarabadi et al. (34)	Holding courses on the empowerment of community health instructors Offering specialty community health nursing programs instead of routine and usual clinical care Reforming nursing curriculum focusing on community-based training Changing educational approaches in community health apprenticeship
	**Community health nursing practice**
Karimi et al. (1), Barasteh et al. (29), Mofrad et al. (38), Yuan et al. (9), WHO (6), Heydari et al. (16)	Revising and reforming the community health nurses' recruitment process Developing an organizational title for the recruitment of health nurses at the community level Formulating the job descriptions and the roles of community health nurses
	**Policymaking**
WHO (6), Heidary et al. (2)	Involving community health nurses in the issues related to national health Formulating laws in support of community health nurses
	**Management**
Ranjbar et al. (30), Yazdani et al. (21), Yuan et al. (9), Oros et al. (45), WHO (6), Jamshidi et al. (46)	Supporting community health nurses for the establishment of community health clinic Using motivational strategies for attracting community health nurses Explaining the concept of the community health nurse and its capabilities
	**Infrastructures**
Oros et al. (45), Heydari et al. (16), WHO (6), Jamshidi et al. (46)	The insurance coverage of community-based care Strengthening inter-sectoral and interdisciplinary cooperation
	**Culture**
Heydari et al. (16), Heydari et al. (18)	Introducing the community health nurses' role and promoting public awareness of it
	**Research**
Kemppainen et al. (13), Yuan et al. (9), Heidary et al. (2)	Investigating the effectiveness of health nursing in the public health promotion

#### The Challenges of Community Health Nursing in Iran

**Figure 2:**
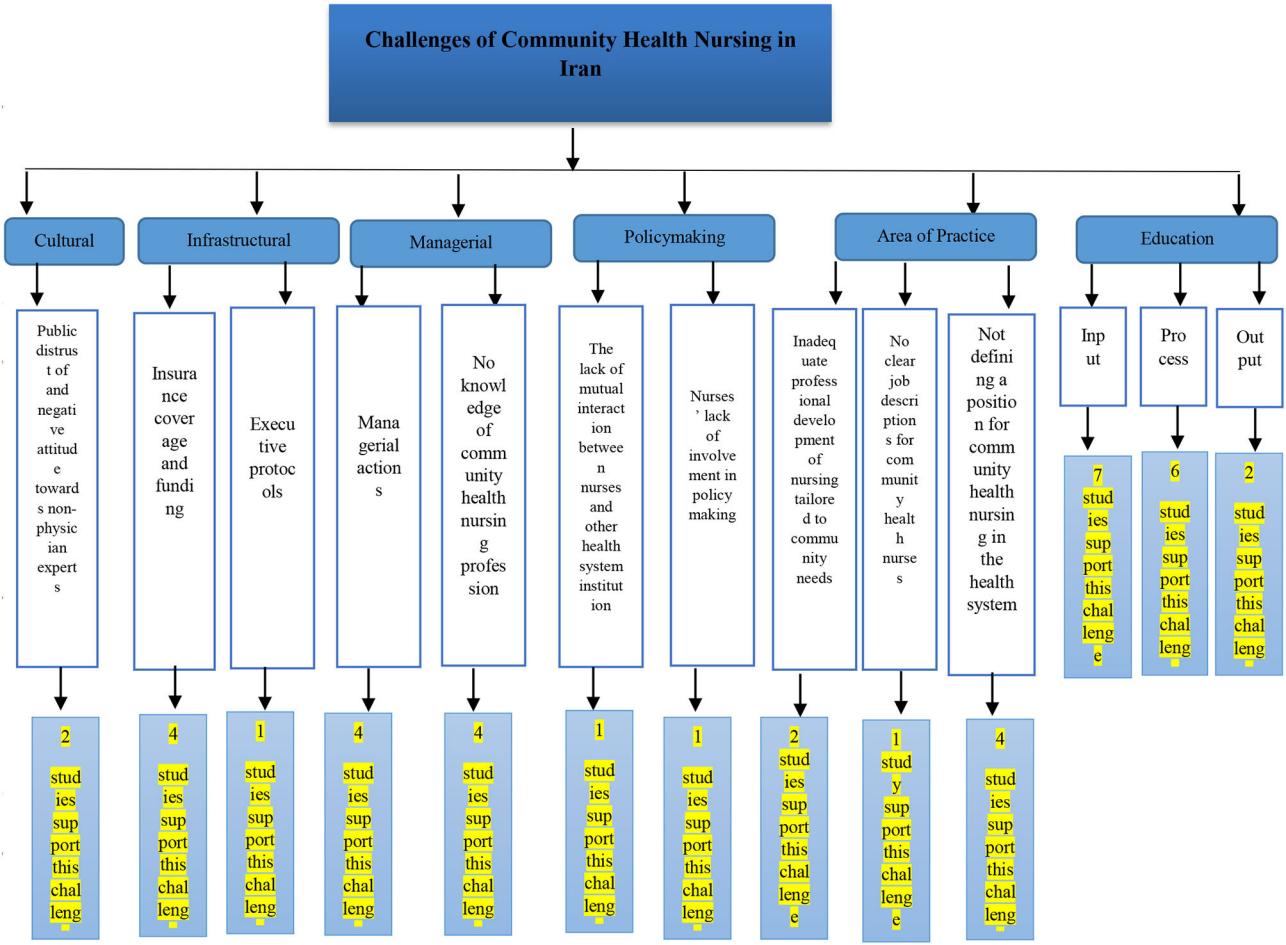
Community health nursing challenges in Iran.

There are several challenges regarding community health nursing in Iran, which have been addressed in previous studies (Figure 2)

##### Challenges of Community Health Nursing Education

Several challenges were mentioned in the literature in regard with the education, which were divided into the three areas *input, process*, and *output*.

###### Input.

Students' poor understanding of community health nursing lessons, considering the community health nursing apprenticeship as futile, and, in some cases, even as a chance to rest, as well as their poor motivation for active participation were mentioned as important challenges (32). Moreover, nursing students' treatment-based and disease-based perspective and their poor community-based and holistic perspective is another challenge in this area (1, 2).

Another challenge in this area is the insufficient skills and experience of nursing educators in the field of community-based educational planning, their inadequacy in conducting community health apprenticeship programs and the related evaluations, and ineffectiveness of community health apprenticeship (19, 42). In another study, the low quality of community health nursing education, the use of traditional methods, reviewing theoretical topics during apprenticeship, and not implementing appropriate educational models were mentioned as challenges (1, 19). Other studies have shown that the educational system of medical universities is not adjusted to PHC and the educational content is not tailored to the needs. Therefore, the university graduates do not have the required skills to deal with problems and academic education courses should be promoted and based on PHC (22, 47).

###### The Process.

The poor presence of community-based care in the nursing education and focusing on hospital care have been referred to as one of the most important challenges according to several studies. Nursing education in Iran is more focused on clinical education. Most nursing schools train their students to play the traditional nursing role, while community needs the training of nurses according to holistic perspectives (1, 16, 19, 40). Limited community health credits and the hours of apprenticeship in health centers will lead to poor productivity of apprenticeship programs (2, 31). The theory-practice gap, i.e., the inapplicability of some health theory content in the apprenticeship settings, and the lack of community-based education standards in nursing were mentioned as other challenges in this field (1, 19).

###### Output.

Recruiting nurses only in clinical settings such as hospitals and the absence of a particular and appropriate professional position for nursing and community health nursing graduates in health centers has prevented nursing graduates from acquiring the necessary skills to provide health care (31, 42).

##### Practical Challenges of Community Health Nursing

One of the most important challenges in this field is not defining positions for the provision of nursing services in the community and various settings of the health system. Therefore, in hospitals, which are considered as the main position of nurses, appropriate roles are not defined for them with the aim of health promotion (21). Another challenge is hiring nurses and community health nurses in medical settings and hospitals. Nursing care in Iran focuses on the provision of care at the secondary level of prevention; therefore, the preventive role of nurses is overshadowed and nurses are not involved in health care homes and comprehensive health centers, which is the first level of people's contact with the health care system (16, 31, 40). The lack of adequate and proper health care in remote and rural areas is one of the biggest national concerns, and most of the health care workers in rural and remote areas are Behvarzes (rural health workers) and practical nurses, while no job opportunities exist for community-based nursing postgraduates in comprehensive health centers, prevention units, and health care homes (2). The lack of clear job descriptions for community health nurses and community-based nursing in the country is another challenge, as community health nursing is only a field for study, not suited for practice (18). Another important challenge will be the inadequate professional development of nursing compatible with the needs of community, the lack of competent staff in the field of community-based nursing, and the lack of nursing promotion in accordance with the pattern of diseases in the country (29).

One of the important areas of community health nursing is performing home visits and the provision of care and counseling in the home environment. A challenge that community health nurses face in this regard is the problems with ensuring their safety (16).

##### Policymaking Challenges in Community Health Nursing

The lack of mutual interaction between nursing and other institutions of the health system and nurses' lack of involvement in policymaking regarding their own field of study has resulted in neglecting the role and the position of nurses in the health system. On the other hand, health policy makers have not taken the nursing profession seriously because physicians are dominant in the health system. In other words, the nurse's role and position in the field of prevention and community-based services has been ignored. The chaos and the disorder in the health system, as another challenge, has prevented the fulfillment of one's actual

role (21). On the other hand, the absence of interaction between nursing managers and the policymakers of the Ministry of Health to identify community health nurses' potentials is another challenge (31).

##### Managerial Challenges in the Field of Community Health Nursing

In the field of management, several challenges have been mentioned in the articles, which were divided into two areas: *managerial actions*, and *the lack of knowledge of the community health nursing profession*.

###### Managerial Actions.

The lack of an effective management system for the use of community health nursing services has been mentioned as one of the most important challenges, as health managers have to make appropriate strategic plans (18). Another important challenge mentioned in the studies is the existence of specialty and subspecialty service providers in the health system, which has hindered the establishment and the development of health promotion centers; in other words, community-based care has no place in the Iranian health system (16). The managers' not using motivational mechanisms to encourage community health nurses to work in health centers is referred to as another challenge. One of the major motivating factors is the allocation of salaries and financial benefits, but since the health personnel's salaries are lower than those of the staff in treatment sectors, community health nurses are less motivated to work in the field of health (2, 31).

###### The Lack of Knowledge of Community Health Nursing Profession.

One of the important challenges is nursing institutions' lack of knowledge of nursing and its specialty disciplines (40). The health managers' lack of knowledge of the capabilities of nursing and community health nursing has resulted in their limited presence in community health service centers and the concept of community health nurse's role (2, 31, 40). In addition, Iranian health managers have not considered a position for community health nurses in the family physician team, because these authorities do not have any knowledge of the community health nursing profession (18).

##### Infrastructural Challenges

In regard with of infrastructure, several challenges were pointed to in the articles, which were divided into three areas: *insurance coverage and funding, executive protocols*, and *interdisciplinary cooperation*.

###### Insurance Coverage and Funding.

Another challenge is the high cost of health service provision in the community in the form of home visits and the fact that they are not paid by insurance. Allocating less funds for providing community-based care than for treatment care and hospitalization is another challenge in the Iranian health system (16, 17, 31, 33).

###### Executive Protocols.

The absence of clear guidelines for making assessments, classifying patients and care seekers, wages and salaries, allocating funds and determining personnel adequacy and competency in the community-based care and home care system is another important challenge in this regard (16).

###### Interdisciplinary Cooperation.

There is no interdisciplinary cooperation and coordination among different sectors of community such as health centers, municipalities, and the police to provide community-based care by community health nurses (16). While the desirable future of the community health nursing profession requires cooperation and communication with other institutions such as the Welfare Organization, the municipality, and the Broadcasting Corporation (29). On the other hand, Iranian health system is governed through unionism and tribalism. Therefore, as long as there is unionism, no interdisciplinary cooperation and partnership should be expected (31).

##### Cultural Challenges

Public distrust of and negative attitude toward the capabilities of non-physician experts in providing prevention and health services is another challenge in Iran (2, 16).

#### Solutions

In previous studies, several strategies were mentioned for each group of community health nursing challenges.

##### Appropriate Solutions to the Challenges of Community Health Nursing Education

###### Input.

In order to solve the challenges in this area, the following strategies can be used:

1. A solution to instructors' challenges: one strategy is to hold training courses for community health instructors so that they can provide and train quality nursing services outside the hospital. It is also possible to provide information on new educational strategies, novel learning opportunities in the field of community health, new tools, and new methods of student assessment to the community health nursing instructors in workshops. In addition, qualified instructors should be hired to teach community health, and short-term training programs should be developed and prepared in the field of geriatric nursing, school nursing, occupational nursing, community-based rehabilitation, and behavioral disease counseling for senior community health nursing professionals in order to promote nurses' position and role in the educational, treatment, and health care teams (1, 2, 19, 43, 44).
2. A solution to students' challenges: the strategy is to hold pre-teaching workshops in various fields of community health with the aim of preparing students to share their apprenticeship goals and motivate them. Furthermore, to train nursing students for community health nursing discipline, participatory processes with key stakeholders such as health centers and deputies, regional hospitals, clinics and schools as well as specialty nursing programs should be used to empower nurses, instead of providing conventional and routine clinical trainings (1, 6, 16).

###### Process.

Modifying nursing curriculums with the aim of focusing on community-based education, developing evidence-based curriculums based on the health needs of different regions of the country, and considering community health nursing practice is an effective strategy (1, 2, 29). Measures can be taken to overcome challenges in this area through changing teaching methods in apprenticeships (considering working with the community and family instead of the individual) and using nursing theories, including Betty Neuman's theory in community health education, the main purpose of which is comprehensive and continuous patient care and, accordingly, the actual position of nurses in care is all the three levels of prevention. We can take steps to address the challenges in this area (1, 42). Using multiple approaches, and a combination of different methods in community health nursing apprenticeships has been effective in increasing nursing students' competence. Training courses in the field of community-based visits (home, schools, factories, and stores) had a positive effect on changing students' attitudes toward community services (34).

##### Appropriate Solutions to the Practical Challenges of Community Health Nursing

This group of challenges can be addressed by reviewing and reforming the process of hiring community health nurses and creating an organizational title for hiring community health nurses in schools, factories, prisons, and comprehensive health centers (1). In other words, this challenge can be overcome by shifting the health care delivery setting to the community, integrating nursing services into primary health care and focusing on health promotion and disease management (29, 38). In addition, reviewing the educational needs of community health nursing graduates to provide ongoing responses to public health needs, especially plans to improve the skills and the abilities of nurses, is one of the effective solutions to this group of challenges (46). At large scale, nursing managers should formulate the job descriptions, roles, and responsibilities of community health nurses (6, 9, 16).

##### Solutions to Policymaking Challenges in Community Health Nursing

This challenge is possible to be solved by involving community health nurses, as professionals in this field, in health-related issues at large scale. Policymakers should also publicize laws and policies in support of community health nurses and develop guidelines that align community health nurses' roles with their skills and areas of practice (1, 2, 6, 26). It is suggested that legislators prioritize accountability principles based on community, justice, and accessibility. In addition, policymakers need to develop a strategy to reform policies in order to lay the ground for the development of nursing at the community level (33).

##### Solutions to Managerial Challenges in Community Health Nursing

Health and treatment managers should support community health nurses in establishing community health clinics (1, 6, 21, 30). Nursing managers should also take action to set up home visit centers using a health promotion approach with the help of community health nurses (33, 46). Nursing managers should take the necessary measures to explain and promote the concept of *community health nurse* and its capabilities (46). Furthermore, health system managers should use motivational strategies to attract and retain competent community health nurses (6, 9, 45).

##### Solutions to Infrastructural Challenges in Community Health Nursing

Nursing managers should provide appropriate guidelines and instructions for community-based care and its management and the insurance coverage of home and community based care and services, as is done in developed countries, which results in more people seeking this type of service from community health nurses (6, 9, 16, 45). Necessary measures should be taken to increase inter-sectoral cooperation between the nursing profession and different fields of community in order to progress, address global health objectives, and strengthen interdisciplinary cooperation (46).

##### Solutions to the Cultural Challenges of Community Health Nursing

Strategies that are effective to address this challenge include informing the community and raising public awareness of the significance and the role of nurses in providing and offering community-based services through social media, as well as developing comprehensive supportive programs in collaboration with the Nursing Deputy of the Ministry of Health, Iranian Nursing Organization, and the National Broadcasting Corporation in order to raise public awareness and understanding of community health nursing (16, 18). Moreover, some studies have proposed a research approach to address the challenges in various fields of community health nursing. Some examples are conducting research to evaluate the effectiveness of community health nursing approach and determine the position of nursing in the health care system, or doing more research on the services provided by community health nurses to make the other members of the health team acquainted with their activities (2, 9, 13).

## Discussion

The aim of this study was to investigate the challenges of community health nursing in Iran and the related solutions in an integrated manner. According to this study, the main challenges included the challenges of community health nursing education, practical challenges in community health nursing, policy-making challenges in community health nursing, managerial challenges in community health nursing, and infrastructural and cultural challenges.

It was discovered that the factors related to community health nursing education are among the challenges. Since health care delivery to people has shifted from hospitals to community centers, nursing students should be educated through community-based approaches. The results of a study on the experiences of nursing instructors showed that the practical training of students in the field of community health is not compatible with the needs of community and education is not community-oriented (48). According to other studies, although one of the goals of community health nursing education is community-based education, it is currently forgotten and ignored (1, 49). The results of the study by Oros focused on education through community-based care models instead of using traditional health models (45). The results of a systematic review study also showed the impact of the effective training and the preparation of nurses in the practical fields of community health to overcome the challenges of community health nursing (24). In their study, Jarrín et al. (50) stated that launching a community health nursing education program in the form of lectures in the first months of university, introducing textbooks, and performing simulations regarding community and home care will significantly affect their attitudes toward and beliefs about community-based nursing because traditional curriculums has resulted in the students' considering community activities and home care unimportant.

The approach through which nurses have been educated during the 20th century is not tailored to the health care needs of the health system in the 21st century. Therefore, nursing educators and planners should constantly review the content of community health nursing education based on the needs assessment of community health students and graduates and according to the needs of the community (37, 44). The study by the World Health Organization also considered the lack of educational standards for community health nursing as a challenge and referred to the need to develop programs in accordance with the educational needs of community health nurses (6). Kemppainen et al. (13) showed that continuing education in accordance with the needs of nurses is effective in their performance in the field of health promotion. Another challenge in this area is the community health nursing instructors' inadequate skills and experiences in providing effective education. In another study, Khorasani reported that the training and nursing courses for nursing students in the field of community health nursing are more focused on filling out care seekers' medical records and collecting health statistics and reports, which are usually done by midwifery or primary health technicians. However, it is less focused on issues such as establishing close relationships with the community and especially families in community health apprenticeship and pays more attention to reviewing theoretical content and superficial visits (51). In other studies, students considered community health apprenticeship futile and useless and believed that there is a relative relationship between theoretical education and the practical field of community health nursing (52, 53).

Challenges related to the field of community health nursing practice are also very important. One of the challenges in this dimension is the lack of competent workforce in the field of community-based care. This finding is in line with the study of Kemppainen et al. (13). One of the reasons behind this is the lack of job opportunities for them to acquire the necessary skills and the lack of retraining courses, while in developed countries like Denmark, nursing graduates are able to make assessments and planning, perform prevention, treat all patients and provide community-based care (54). Another challenge in the practical field of community health nursing in Iran is the lack of a clear job description for community health nurses. In the study by the World Health Organization, disagreement on community health nursing performance was mentioned as one of the important challenges (6). In their study, Kemppainen et al. (13) considered the lack of clarity in the nurses' job descriptions and activities in the field of health promotion as a barrier. In another systematic review study, the lack of a clear definition of the nurses' role in PHC has been mentioned as an important challenge in achieving universal health coverage (24).

Another challenge lies in the field of community health nursing policymaking. What was mentioned in the studies shows the impact of the structure and policies of the health system on not creating an appropriate position for community health nurses in the field of community-based and community-oriented health services. Many countries have given nurses the opportunity to provide primary health care to develop the quantity and the quality of health care in their communities. At present, in the Iranian health system, changes are taking place without considering the needs of care seekers and the costs of health care increase with the rise in the elderly population and the higher percentage of chronic diseases in the community (23). A study conducted in Oman also showed that community health nursing is not considered as much important as hospital-based nursing by the policymakers (55). In the study by Yuan, the lack of community health nurses' participation in health policymaking and planning is one of the major challenges in providing community health nursing services (9). According to the WHO, one of the most important challenges is the lack of public commitment and the policymakers' incapability to execute regional and global policies for community health nurses (6).

Another important challenge is management. One of the challenges in this area is the lack of motivational mechanisms for encouraging community health nurses. In a WHO study, the environment and the non-supportive conditions of community health nurses are stated as challenges and the use of motivational strategies to attract and retain competent community health nurses is regarded as a solution (6). The study of Kemppainen et al. (13) showed that in the organizational culture, the presence of health managers who support nurses in providing health services is an effective factor in nurses' health promotion practice. Another challenge in this area is the health system managers' lack of knowledge of community health nursing, which is consistent with the study conducted by WHO (6). Therefore, considering the emergence of global health threats, it seems necessary to clarify the concept of community health nursing.

Infrastructural challenges are another type of challenge according to this study. Currently, the focus of the health system on disease-oriented approaches rather than prevention is an important reason for the huge amount of money spent in the treatment sector, and causes issues in the allocation of resources to the community health nurses in the field of health promotion. The results of the study by Yuan also regarded the lack of sufficient funding for the provision of community-based nursing services as a significant challenge (9). Moreover, an Omani study reported community health nurses' limited access to equipment and lower funding for implementing the program (55), which is in line with the study of Kemppainen et al. (13). Other studies in other countries have shown that the amount of nurse salaries at the community level and in the society is lower than in medical centers, and these underpaid nurses who provide services at the community level are pushed to work in the treatment sector (56, 57). Another challenge in this area is poor interdisciplinary cooperation. The World Health Organization also considers poor interdisciplinary cooperation with other professions as one of the major challenges of community health nursing (6). Investing in interdisciplinary teamwork is considered as a way to overcome the challenges of nursing in providing primary health care (24).

Cultural issues are regarded as another important challenge. People's distrust of non-physician experts' capabilities is a challenge in this area. A Chinese study also showed the lack of public trust in community health nursing services (9). However, according to the study by the WHO, non-physician staff are also able to provide similar care to patients (58). The findings of another systematic review study aimed at investigating the impact of replacing physicians with nurses in PHC in the care procedure, patient outcomes and cost analysis showed that the care provided by nurses had similar or better health outcomes compared to the care provided by physicians (36). According to the WHO, if the health system wants to address the health needs of the community, it must use nurses and midwives (59). The existence of a communication channel for community health nurses is essential to raise public awareness through the media (47). The study of Heydari et al. (18) also emphasized on preparing the community and raising public awareness in order for them to receive community-oriented nursing services. Therefore, it is necessary to explain the important role of nurses in providing health services to the public.

Considering that all the challenges mentioned in these studies can also be applied to Iranian community health nursing in Iran, it is possible to take an important step toward solving these challenges in the country's health system by implementing the proposed strategies.

### Limitations

This integrated review has several limitations. The authors' knowledge of the challenges of community health nursing and the solutions is limited to the data documented in the articles. Therefore, if the challenges are not fully described or reported by the authors, they will not be reflected in the results. Only credible articles in English and Farsi were reviewed; as a result, the articles and the related data from initial research and gray literature published in other languages may have been omitted. The authors did not seek to evaluate the quality of the studies and did not compare them with similar ones. However, our integrated review is an attempt to combine the results of the studies and the research approaches. Finally, these results are more related to community health nursing challenges in Iran. Future research should address the challenges of community health nursing at a global level.

## Conclusion

Considering the results of the present study, it can be concluded that the challenges of community health nursing in Iran, including the lack of an appropriate position for community health nursing, nursing education ignoring community-oriented care, and inappropriate infrastructure are inter-wound issues dependent on each other that affect community health nursing practice. Therefore, in order to solve these challenges, it is suggested that the policy makers and the managers of the health system modify the structure of the health system so as to move from a treatment-oriented approach toward a community-oriented one, develop supportive laws and job descriptions for community health nurses, and create an organizational chart for community health nurses at the community level. It is also recommended to use motivational strategies to attract community health nurses and support them in establishing community health clinics, and to cover community-based services and care under insurance. Moreover, nursing education administrators should modify nursing students' curriculum with the aim of focusing on community-based education. In addition, in order to solve the challenges in this field, community health nursing leaders should try to increase cross-sectoral and inter-professional cooperation, promote the profession, and make the capabilities of community health nurses recognized by other professions and the public.

## Data Availability Statement

The original contributions presented in the study are included in the article/supplementary material, further inquiries can be directed to the corresponding author.

## Author Contributions

The data analysis and manuscript were prepared by AH with support from SJ, MR. All authors critically reviewed and contributed to the manuscript and approved the final version.

## Funding

This article was a part of a nursing PhD dissertation approved by Ahvaz Jundishapur University of Medical Sciences (No. 1398.874) which was financially supported by the Nursing Care Research Center in Chronic Diseases of Ahvaz Jundishapur University of Medical Sciences (NCRCCD-9837).

## Conflict of Interest

The authors declare that the research was conducted in the absence of any commercial or financial relationships that could be construed as a potential conflict of interest.

## Publisher's Note

All claims expressed in this article are solely those of the authors and do not necessarily represent those of their affiliated organizations, or those of the publisher, the editors and the reviewers. Any product that may be evaluated in this article, or claim that may be made by its manufacturer, is not guaranteed or endorsed by the publisher.
